# Analysis of bus passenger comfort perception based on passenger load factor and in-vehicle time

**DOI:** 10.1186/s40064-016-1694-7

**Published:** 2016-01-22

**Authors:** Xianghao Shen, Shumin Feng, Zhenning Li, Baoyu Hu

**Affiliations:** School of Transportation Science and Engineering, Harbin Institute of Technology, No. 73 Huanghe Road, Harbin, 150090 Heilongjiang People’s Republic of China

**Keywords:** Public transport, Comfort perception, Passenger load factor, In-vehicle time

## Abstract

Although bus comfort is a crucial indicator of service quality, existing studies tend to focus on passenger load and ignore in-vehicle time, which can also affect passengers’ comfort perception. Therefore, by conducting surveys, this study examines passengers’ comfort perception while accounting for both factors. Then, using the survey data, it performs a two-way analysis of variance and shows that both in-vehicle time and passenger load significantly affect passenger comfort. Then, a bus comfort model is proposed to evaluate comfort level, followed by a sensitivity analysis. The method introduced in this study has theoretical implications for bus operators attempting to improve bus service quality.

**Electronic supplementary material:**

The online version of this article (doi:10.1186/s40064-016-1694-7) contains supplementary material, which is available to authorized users.

## Background

Passenger comfort is an important index that can be used to measure the quality of public transport services and a crucial factor in residents’ choice of traffic mode (Dell’Olio et al. 2011; Eboli and Mazzula 2011). For example, the quality of life in China has been increasing over the years, which in turn has led to the demand for higher levels of trip comfort. Presently, traffic congestion has become ubiquitous in China’s metropolitan areas. Thus, improving bus comfort to attract more passengers and further alleviate traffic congestion has received much attention from bus operators and authorities (Zhang et al. 2014). In addition, elucidating the factors affecting bus comfort levels can help policymakers implement targeted improvement strategies.

Lai and Chen (2011) indicated that the perceived value determined by service quality positively affects overall satisfaction, involvement, and behavioural intentions. Comfort is one of the key factors leading to high service quality and significantly influences passenger satisfaction with bus transits (Eboli and Mazzulla 2007, 2009).

Research on bus comfort can be roughly divided into two categories. The first comprises studies on vehicle performance and running status, insofar as they affect passenger comfort (e.g. vibrations (Sekulic et al. 2013), acceleration, jerk magnitude (Castellanos and Fruett 2014), and vehicle noise (Zhang et al. 2014); and the second type includes bus-operating environments, which is the focus of the present research.

Many researchers have studied and proved the importance of passenger load in determining bus comfort. For instance, Vovsha et al. (2014) conducted a survey and showed that when a passenger has a less than 40 % probability of getting a seat, he or she feels uncomfortable. Kumar et al. (2004) estimated the comfort perception of rural bus passengers under three travel conditions—seating, standing comfortably, and standing in a crowd—and showed that comfort perception significantly impacted the generalized cost of passengers. Crowding affects not only physical comfort but also psychological issues, such as anxiety, stress, and feelings of one’s privacy being invaded; in fact, varying crowding levels between competing routes and unbalanced vehicle loads are found to affect passengers’ choice of route and vehicle (Li and Hensher 2011; Tirachini et al. 2013).

Eboli and Mazzulla (2010) studied the effects of passenger load on choice of travel mode using rating and choice options; however, they considered only two levels of bus crowding: overcrowded and not overcrowded. Litman (2008) proposed that when a bus offers a comfortable riding environment, passengers’ perceived journey time is less than the actual journey time. However, the author did not examine whether passengers’ actual journey time affects comfort perception.

In sum, although the extant literature on comfort is extensive, it largely concentrates on the influence of passenger load on comfort levels. More specifically, research attention devoted to in-vehicle time, which may also influence passengers’ comfort perception, is limited, especially in the context of quantity measurements. Although there are other factors affecting bus comfort (Eboli and Mazzulla 2007; Shek and Chan 2008), this study focuses on passenger load and in-vehicle time.

Passengers’ judgments about certain service attributes can be considered a subjective measure of service quality, while performance measures contingent on bus operators can serve as objective measures of service quality (Eboli and Mazzula 2011). Li and Hensher (2013) suggested that, in addition to using objective measures (e.g. passenger load), bus operators and authorities should conduct perception surveys to obtain information on passengers’ subjective evaluation of bus services (e.g. bus comfort).

Thus, precisely evaluating passengers’ perception is a necessary prerequisite to improve bus comfort. This study, therefore, conducts surveys on perceived comfort evaluation using two objective factors, passenger load factor and in-vehicle time, to examine the real experiences of passengers about ride comfort. The findings of these surveys can help bus operators and authorities design more appealing measures to improve bus comfort level.

## Methods

### Survey design

Passengers’ perception is an important criterion in evaluating the level of bus service quality as well as other objective performances (De Ona et al. 2013). To obtain the value of passenger comfort perception along with in-vehicle time under different passenger load factors, a two-day passenger comfort perception survey was conducted on July 31 and August 1, 2014, at Bus Line 63 in Harbin City, China. A total of 10 investigators from the Harbin Institute of Technology (HIT) who regularly used the bus line participated in the survey as the passengers. Line 63 is representative of most bus lines in the city in terms of vehicle size, with the rated passenger capacity of 80P, and has obvious passenger flow characteristics during rush and off-peak hours.

On the basis of local language and manners of expression in China, comfort perception in the survey scheme is divided into five grades and accordingly, assigned scores (Table 1). Table 1 draws on the method of a Likert scale for reference, and the expressions of perception were fully communicated with the investigators to ensure clear judgment on the basis of the table. A higher score denotes a greater comfort level. If passenger perception falls between two levels, its corresponding score takes the average of the two scores; for example, if perception falls between ‘comfort’ and ‘slightly uncomfortable’, its corresponding score is 6. Second, according to the different passenger load factors, congestion is divided into five grades (Table 2). Each grade is depicted on the basis of the observed condition using a real in-vehicle survey and corresponding passenger load factors. In some countries, passenger load factor is defined as the ratio of the actual number of passengers in a vehicle to the number of seats; however, in China, the number of seats is always significantly less than that of passengers, and thus, in this study, passenger load factor is defined as the ratio of the actual number of passengers to the rated passenger capacity of the bus in Table 2 (Liu and Li 2008).

**Table 1:** Comfort perception level and scoring criteria

Perception level	Extremely uncomfortable	Very uncomfortable	Slightly uncomfortable	Comfortable	Very comfortable
Scoring criteria	1	3	5	7	9

**Table 2:** Congestion level classifications

Congestion level	Passenger load factor	Illustration
1	0.35	Everyone on the bus has a seat
2	0.50	With a relaxed riding environment, the distance between two standing passengers is at least the width of one person
3	0.60	Slightly crowded, there is no body contact between standing passengers, although spacing is very close, and when moving there will be incidental body contact
4	0.75	Crowded, there is slight body contact between standing passengers; sudden braking or cornering would cause greater contact
5	1.00	Very crowded, with significant body contact between passengers

The survey method is as follows. First, we conducted an in-vehicle examination for the 10 investigators to record the passenger load factor and evaluate the comfort scores of their own perception at 5-min intervals. Each investigator takes four trips on the bus line: two trips in the morning rush hour and the other two during off-peak hours in the afternoon; each trip lasts about 25–40 min. In this case, the investigators experience varying passenger load factors and in-vehicle time. However, the passenger load factor tends to vary by station and thus, the investigators do not experience the level for a long time. Therefore, using the in-vehicle survey in combination with the digital images depicting varying congestion levels, the investigators judged their own immersive perception to evaluate bus comfort. An example with a passenger load factor of 0.5 is shown in Table 3.

**Table 3:** Comfort perception evaluation example when the passenger load factor is 0.5

In-vehicle time (min)	5	10	15	20	25	30
Seated	9	9	9	9	8	8
Standing	7	7	6	6	6	5

Line 63, which connects the two HIT campuses, is frequently used by the 10 investigators. Thus, they are required to combine their past feelings when evaluating comfort perception to avoid survey affects on their real perceptions.

On both survey days, the temperature was 29–30 °C and the weather was sunny. The surveyed bus was not air-conditioned but clean. This is the first time that this kind of comfort perception survey conducted in the city.

Using the survey, we were able to collected 540 comfort perception samples from the 10 investigators, which included 300 seated samples and 240 standing samples. A passenger load factor of 0.35 denotes that each passenger was able to get a seat; thus, we did not evaluate the comfort perception of the standing sample at this level, which explains the lower number of standing samples than seated ones (Additional file 1).

### Analysis of variance for comfort perception

To examine the effects of passenger load factor and in-vehicle time on passenger comfort perception, we conducted a two-way analysis of variance (ANOVA) under two circumstances (seated and standing) with a significance level of α = 0.05. We accounted for five passenger load factors and six in-vehicle time levels. The descriptive statistics of the survey data (Table 4) show that seated and standing passengers perceive near-comfortable and slightly uncomfortable feelings, respectively. Table 5 presents the results of the two-way ANOVA for three variables: in-vehicle time, passenger load factor, and both. The asterisk in the table denotes interaction of the two variables. The former two account for substantial effects and third for interaction effects. As shown in the table, the p value of the substantial effects is much lower than 0.05, indicating a significant effect of both passenger load factor and in-vehicle time; however, no interaction effects of the two factors were found.

**Table 4:** Descriptive statistics

Statistic	N	Mean value of comfort perception	SD
Seated	300	6.48	1.83
Standing	240	4.25	1.90

**Table 5:** Two-way ANOVA for comfort perception

Statistic	Independent variable	Sum of squares	df	Mean square	F	Sig.
Seated	In-vehicle time	90.137	5	18.027	15.046	0.00
	Passenger load factor	589.387	4	147.347	122.979	0.00
	In-vehicle time* Passenger load factor	1.813	20	0.091	0.076	1.00
Standing	In-vehicle time	123.850	5	24.770	22.884	0.00
	Passenger load factor	500.967	3	166.989	154.275	0.00
	In-vehicle time* Passenger load factor	4.383	15	0.292	0.270	1.00

**Fig. 1:**
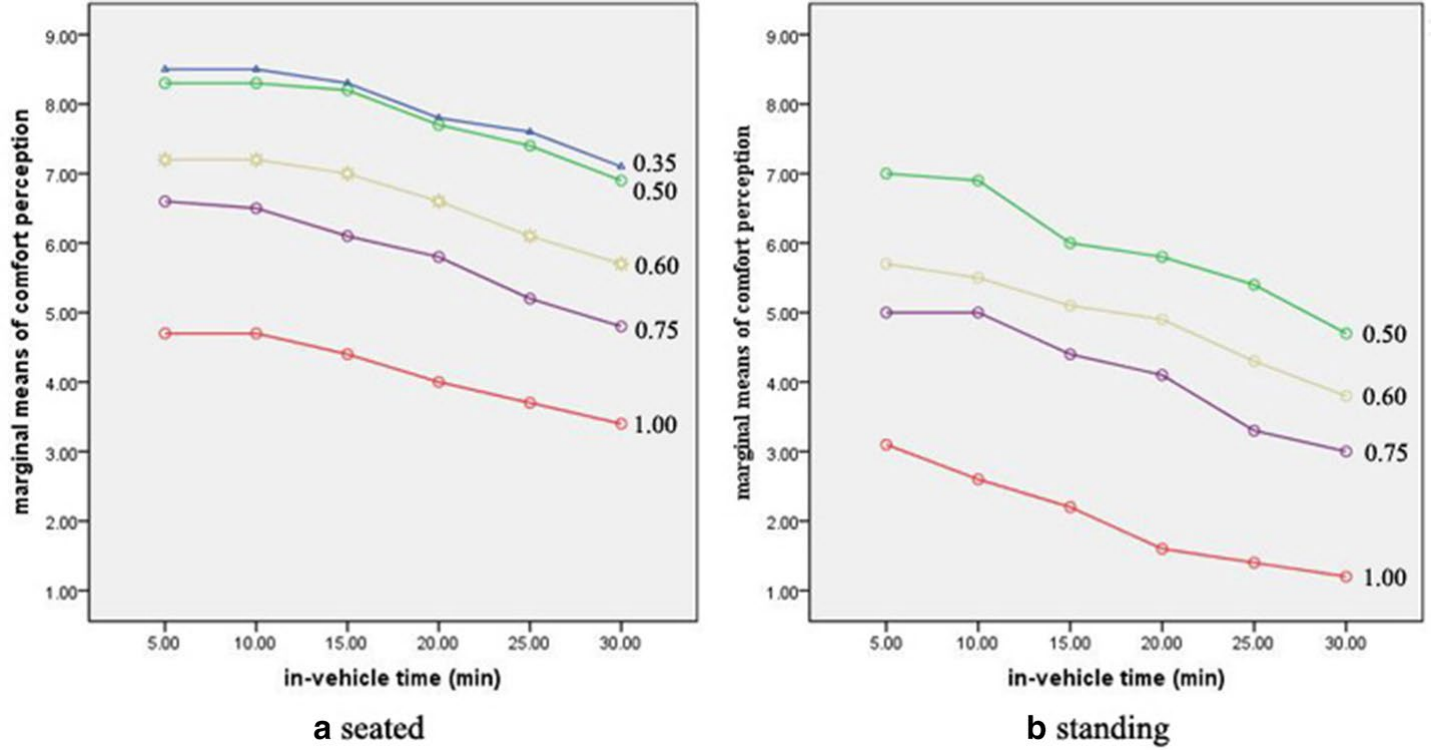
Comparison of marginal means of comfort perception. Figure 1 shows the comparison of the marginal means of comfort perception between being seated and standing and the *number after each line* indicates the passenger load factor

Figure 1 is a comparison of the marginal means of comfort perception between the seated and standing samples, and the number after each line indicates passenger load factor. As can be seen, irrespective of passengers being seated or standing, the comfort perception score decreases with an increase in in-vehicle time and degree of congestion (passenger load factor). The standing comfort perception curve decreases more sharply than the seated curve as in-vehicle time increases to the same level as the passenger load factor.

In general, within the scope of the survey data, the effect of passenger load factor on comfort perception is larger than that of in-vehicle time, especially for seated passengers. However, in-vehicle time also shows a significant effect after 10 min; in particular, the effect becomes stronger for standing passengers as time passes.

### Model construction for bus comfort evaluation

We apply the abovementioned analysis results and consider other factors (e.g. temperature and cleanliness) as fixed on the basis of the characteristics of passenger comfort perception. Bus comfort for a certain period can be evaluated using the average passenger load factor and in-vehicle time and expressed using the following formula:

1 $$CP = a\bar{T} + b\bar{L} + c,$$

where *CP* is a bus line’s average bus comfort value (seated or standing) in a certain period; $\bar{T}$ is the average in-vehicle time of passengers during the period; $\bar{L}$ is the average passenger load factor for the bus line in the period; and *a*, *b*, and *c* are parameters to be estimated. By obtaining the value for *CP*, we determine the comfort level of a bus line according to Table 1.

From the analysis in “Methods” section, under the same passenger load factors and in-vehicle time effects for Bus Line 63 in Harbin City, we find that the comfort perception of seated passengers is higher than that of standing passengers. Thus, we only control for the comfort level of standing passengers. A multiple regression analysis was then conducted to fit the calibration parameters for the 240 standing samples obtained from the survey; the results are shown in Table 6. Equation (2) is the comfort evaluation model for standing passengers on Bus Line 63.

**Table 6:** Multiple regression analysis

Adjusted R-square	Model	Unstandardized coefficients		t	p value	Collinearity statistics tolerance
		B	SE			
0.714	(Constant)	11.151	0.290	38.481	0.000	
	In-vehicle time	−0.084	0.008	−10.897	0.000	1
	Passenger load factor	−7.630	0.348	−21.904	0.000	1

2 $$CP = 11.151 - 0.084\bar{T} - 7.630\bar{L}.$$

The average in-vehicle time of passengers in a certain period can be obtained using the following equation:

3 $$\bar{T} = \frac{{\sum\nolimits_{i = 1}^{n - 1} {I_{(i,i + 1)} \cdot t_{(i,i + 1)} } }}{{\sum\nolimits_{i = 1}^{n - 1} {U_{i} } }},$$

where $I_{(i,i + 1)}$ is the cross-section passenger flow between station *i* and *i* + 1 and is in P, $t_{(i,i + 1)}$ is the time spent between station *i* and *i* + 1 and is in min, $U_{i}$ is the number of passengers who get on at station *i* and is in P, and *n* is the number of stations on the bus line.

The average passenger load factor of a bus line in a certain period can be obtained using the following equation:

4 $$\bar{L} = \frac{R}{Q},$$

where *R* denotes the average cross-section passenger flow of the bus line and *Q* denotes the bus supply of the line and both are in **P.**

*R* can be calculated as follows:

5 $$R = \frac{{\sum\nolimits_{i = 1}^{n - 1} {I_{(i,i + 1)} } }}{n - 1}.$$

Next, taking the morning rush hour and off-peak hours for Line 63 as our study period, we conducted an in-bus survey for two trips; each trip represents the passenger volume characteristics of the corresponding period. The survey recorded the number of passengers that get on and off at each bus station to obtain the cross-section passenger flow between the two stations, the travel time between the two stations, the number of stations (*n* = 21), and the rated passenger capacity (*Q* = 80P). Using survey data, we can obtain the value of relative parameters, as shown in Table 7. Then, using formulae (3–5), we obtain the average in-vehicle time and passenger load factor for the two periods and further calculate the comfort evaluation of the bus line, the results of which are shown in Table 8.

**Table 7:** Relative parameters for passenger volume for Bus Line 63

Time period	$\sum\limits_{i = 1}^{n - 1} {U_{i} }$ (P)	$\sum\limits_{i = 1}^{n - 1} {I_{(i,i + 1)} }$ (P)	$\sum\limits_{i = 1}^{n - 1} {I_{(i,i + 1)} } \cdot t_{(i,i + 1)}$ (P min)	*R* (P)
Morning rush time (7:00–8:00)	116	918	4106	46
Off-peak hours (14:00–15:00)	90	747	1514	37

**Table 8:** Comfort evaluation for Bus Line 63

Time period	Average in-vehicle time (min)	Average passenger load factor	Score of comfort evaluation	Comfort evaluation
Morning rush time (7:00–8:00)	35	0.57	3.86	Close to very uncomfortable
Off-peak hours (14:00–15:00)	16	0.46	6.29	Close to comfortable

### Sensitivity analysis

**Fig. 2:**
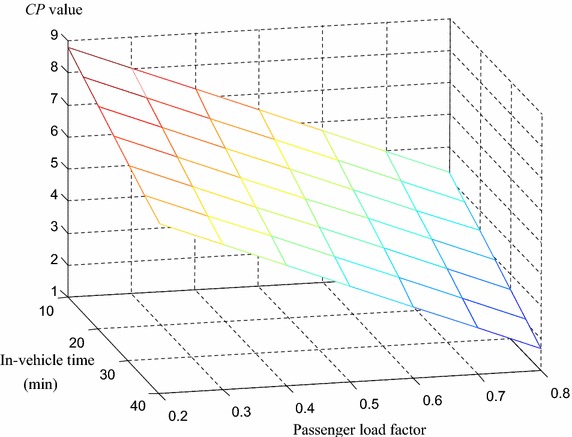
Sensitivity analysis of the bus comfort of standing passengers. By taking Bus Line 63 as an example, conduct a sensitivity analysis for the comfort evaluation of standing passengers under different in-vehicle time and passenger load factors to provide a theory basis for the improvement of bus operation

Using the above comfort evaluation model with Bus Line 63 as an example, we conducted a sensitivity analysis to evaluate the comfort levels of standing passengers under different in-vehicle time and passenger load factors; the findings of this model can provide a theoretical basis for improvements in bus operations (see Fig. 2).

Considering the actual operational situation of Bus Line 63, both average in-vehicle time and passenger load reach the lowest levels before the morning rush hour. From Table 8, we see that the corresponding values during 14:00–15:00 are 16 min and 0.46. With faster bus speeds and fewer passengers at 06:00, we assume these values to be as low as 10 min and 0.2. Similarly, average in-vehicle time and passenger load could reach their highest levels—assumed to be 40 min and 0.8—during the morning or evening rush hour combined with bad driving conditions (e.g. accidents or bad weather); in this case, average bus speed can be lower than 10 km/h.

Because Fig. 2 is created using the comfort model of standing passengers and we assume that the minimum passenger load factor at which some passengers need to stand is 0.4, the maximum *CP* value for standing passengers is greater than 7, indicating that passengers were comfortable. We also see that the minimum *CP* value is less than 2, which means passengers’ perception is close to extremely uncomfortable.

## Results

In-vehicle time considerably affects passenger comfort perception, as does passenger load factor, and has a more significant impact on standing passengers than seated ones.

The evaluation model for bus comfort reflects authentic passenger perceptions and real-life environments, and thus, can serve as a policy basis for bus operations and traffic management.

As Fig. 2 shows, if a bus operator wants to control the bus comfort level around slightly uncomfortable (*CP* = 5) or more, the maximum average passenger load should be 0.7, because when the average passenger load factor exceeds 0.7, despite the average in-vehicle time lowering to 10 min, the *CP* value remains at less than 5. When the average in-vehicle time is 40 min, the average passenger load should be less than 0.36; otherwise, the *CP* value will be less than 5 and passengers may feel worse than slightly uncomfortable.

In sum, relevant authorities should consider the effects of both in-vehicle time and passenger load factor on comfort levels when developing public transport scheduling schemes and traffic management measures.

## Discussion

It is noteworthy that Eq. (2) holds for Bus Line 63 in Harbin City subject to certain conditions—cleanliness, thermal comfort, temperature, and vehicle performance. Equation (2) is also applicable to other bus lines, provided they are similar to Line 63. Further, passengers’ comfort perception may differ between China and Western countries. Because the cost of conducting surveys for each line under different operating conditions is too high, future studies must address the efficient categorization of bus-operating contexts and select an appropriate comfort evaluation model for each context.

The above analysis revealed that to improve passengers’ comfort perception, both passenger load factors and in-vehicle time should be considered and optimized in terms of traffic demand management (TDM), transportation resource allocation, and bus operations (Daganzo 2010; Meyer et al. 1997).

TDM is an effective way of reducing the use of private cars, which in turn can significantly alleviate congestion and thus, improve bus speed and comfort as well as increase the turnover rate. It is noteworthy that with a TDM policy, more passengers will choose buses as their mode of transport, resulting in higher passenger load. Thus, the government should ensure sufficient bus supply using rational transportation resource allocation.

Transportation resource allocation should give priority to public transportation. Improving bus speeds by constructing more bus lanes and setting up bus priority signals are of key strategies. In addition, reward and punishment mechanisms in the form of financial subsidies should be implemented; this may prompt bus operators to increase bus supply, thus reducing passenger load and increasing passenger comfort levels.

Finally, bus operations should make optimum utilization of bus line networks, such that passengers can reach their destinations with a minimum non-linear coefficient. In addition, extreme weather conditions such as heavy snowfall can lead to prolonged in-vehicle time. To tackle these issues, the comfort evaluation model suggests that operators should increase bus frequency, maintain a certain level of riding comfort, and improve service quality.

In the comfort perception model, in-vehicle time is an objective measure; however, the subjective judgement of passengers may differ from objective measures. For example, if buses offer WiFi services, then passengers with smartphones will perceive less in-vehicle time. By contrast, those in a hurry to reach work will perceive longer in-vehicle time. Thus, not accounting for subjective judgement on in-vehicle time is a shortcoming of the model and should be improved in future research. Furthermore, in the model, passengers’ comfort perception is assumed to be a continuum and the discomfort perception is accumulated with an increase in passenger load factor and in-vehicle time; the correctness of this assumption should be validated by research on human factors and ergonomics.

## Conclusions

This research aimed at examining the effects of passenger load factor and in-vehicle time on passengers’ comfort perception. To do so, it used a bus line in Harbin City as a practical example. The main findings are as follows.

Both in-vehicle time and passenger load factor substantially affect passenger comfort perception. To improve and maintain a high level of bus comfort, besides optimization of bus operations, related authorities should implement a TDM policy and optimize transportation resource allocation.

The characteristics of passenger comfort perception studied in this work can help authorities and bus operating companies optimize relevant strategies to facilitate a comfortable transit experience for bus passengers and strengthen the competitive power of public transport.

## Additional file

10.1186/s40064-016-1694-7 Passenger comfort perception survey at Bus Line 63 in Harbin City. The data illustrate comfort scores of the bus line under different levels of passenger load factor and in-vehicle time.
